# Soft UV nanoimprint lithography-designed highly sensitive substrates for SERS detection

**DOI:** 10.1186/1556-276X-9-623

**Published:** 2014-11-21

**Authors:** Maximilien Cottat, Nathalie Lidgi-Guigui, Inga Tijunelyte, Grégory Barbillon, Frédéric Hamouda, Philippe Gogol, Abdelhanin Aassime, Jean-Michel Lourtioz, Bernard Bartenlian, Marc Lamy de la Chapelle

**Affiliations:** CSPBAT (UMR 7244), CNRS-Université Paris 13, 74 rue Marcel Cachin, 93017 Bobigny, France; IEF CNRS UMR 8622, Université Paris-Sud, Bâtiment 220, Rue Ampère, 91405 Orsay, France

**Keywords:** Soft UV-NIL, Nanoimprint, SERS biosensors, Avidin, Biotin

## Abstract

We report on the use of soft UV nanoimprint lithography (UV-NIL) for the development of reproducible, millimeter-sized, and sensitive substrates for SERS detection. The used geometry for plasmonic nanostructures is the cylinder. Gold nanocylinders (GNCs) showed to be very sensitive and specific sensing surfaces. Indeed, we demonstrated that less than 4 ×10^6^ avidin molecules were detected and contributed to the surface-enhanced Raman scattering (SERS) signal. Thus, the soft UV-NIL technique allows to obtain quickly very sensitive substrates for SERS biosensing on surfaces of 1 mm ^2^.

## Background

Surface-enhanced Raman scattering (SERS) technique was shown to be a very effective analytical tool for the detection and identification of molecules, thanks to its high sensitivity [1, 2]. It has been widely used for ultrasensitive chemical analysis down to the single molecule sensitivity. Its field of applications is as varied as to include chemical-biochemical analysis, nanostructure characterization as well as biomedical applications [3–7]. Especially in chemistry, SERS is applied for the detection of conformational changes and structural differences regarding preferred orientations of molecules with respect to a metal surface [8]. The facts that SERS gives a specific fingerprint of a molecule and is sensitive to very small molecules make it a good candidate for application in the fields of chemical and biological sensors. The SERS enhancement is due to the localized surface plasmon resonance (LSPR) of the metallic nanostructure. The nanostructured LSPR properties need to be designed and strongly controlled in order to produce highly reproducible active SERS substrates [9, 10]. In previous studies, we have discussed the necessity to optimize the size of gold nanocylinders (GNCs) in order to achieve the highest possible SERS enhancement. In addition, we have demonstrated the necessity to optimize the LSPR in the case of each studied molecules [11].

For industrial applications, nanostructured surfaces of at least 1 mm ^2^ have to be produced. In the past, electron beam lithography (EBL) has helped us to demonstrate that by optimizing the nanostructure assembly parameter, enhancement factors estimated at 10 ^5^ to 10 ^7^ could be obtained for such proteins as bovine serum albumin (BSA) or RNase-A [11]. However, EBL is expensive and time-consuming. Techniques to produce large and organized nanostructured assemblies on transparent substrates have been developed since several years in order to maximize Raman scattering enhancement [12, 13]. The most popular is probably the nanosphere lithography (NSL) [14]. The advantage of this technique is to obtain large areas (several mm ^2^) of nanostructures on a substrate. However, the shape and arrangement of the nanostructures are more hardly tuned.

In this article, we propose to use another technique called soft UV-nanoimprint lithography (UV-NIL) [15] in order to fabricate SERS substrates. UV-NIL is biocompatible, since it can be implemented on any flat surface. Another essential advantage is that the samples produced with the same mold are all identical. This is important to guarantee the reproducibility of the results. We have already demonstrated the use of UV-NIL for the detection of biomolecules using a LSPR shift [16] and for the realization of nanoholes for AFM studies of membrane proteins [17]. Large arrays of reproducible nanostructures are more and more implemented for SERS [18, 19]; nevertheless, they are rarely used for biosensing. Galarreta et al. have demonstrated from functionalized nanotriangles obtained by NSL the detection of avidin [20]. We can explain this phenomenon by making several assumptions. First of all, the technologies available to produce large gratings of nanostructures on transparent substrates are quite recent and still the domain of physicists. The second point is that for biodetection, the nanoparticles must be functionalized, firstly, in order to preserve the biomolecules from surface interactions and, secondly, to guarantee specific biosensing. Eventually, biomolecules are difficult to handle and have low Raman cross section. In order to determine the properties of our UV-NIL substrates as a SERS sensor and determine its sensing performances, we have chosen to study the biotin/avidin system.

## Methods

### The fabrication of gold nanocylinders by UV-NIL

**Figure 1:**
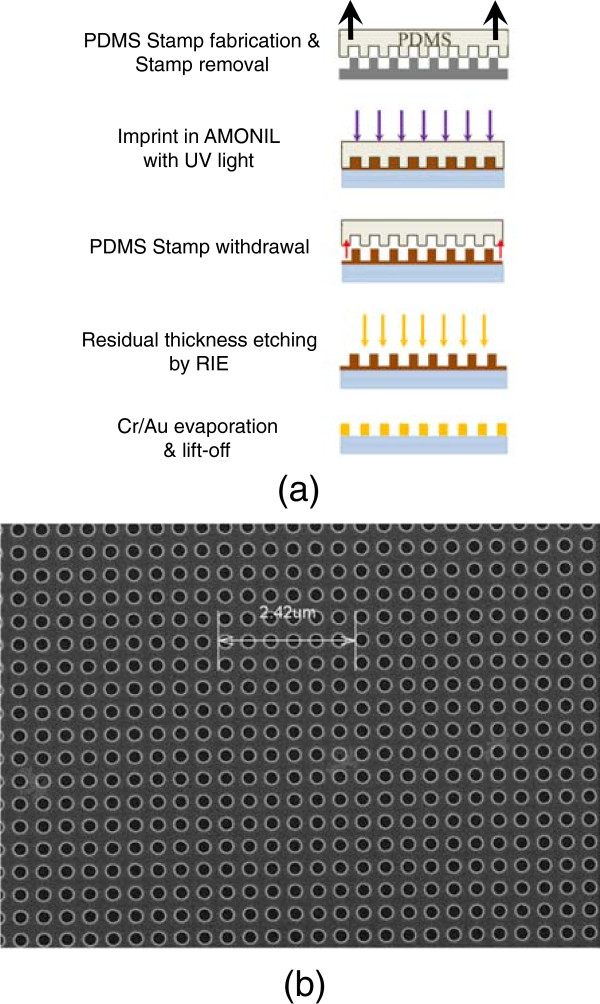
**UV-NIL.** **(a)** Principle scheme of UV-NIL. **(b)** SEM image of Si master mold (diameter of approximately 220 nm, periodicity 400 nm).

Figure 1a displays the main steps of the UV-NIL process. The first step of this technique is to fabricate a master mold. As done in previous studies, this master mold was fabricated using EBL on PMMA resist combined with reactive ion etching for the pattern transfer into the silicon substrate (see Figure 1b). The conditions of this transfer have been published in [17]. The use of a single master mold for all the samples studied here guarantees the reproducibility of the GNCs. The stamps were fabricated with the standard poly(dimethylsiloxane) (PDMS) diluted in hexane solvent in order to reduce the viscosity and thus to improve the penetration of the PDMS in nanoholes [21]. The stamp was pressed against PMMA/AMONIL resists, which were deposited on a glass substrate, using an EVG 620 mask aligner. The addition of solvent in the PDMS stamp also reduces the interface adhesion and allows easy separation between the stamp and the AMONIL resist, which is used for the imprint. We combined this patterning method with subsequent metal deposition and lift-off process. This procedure leads to a very regular grating of GNCs.

### Avidin detection

**Figure 2:**
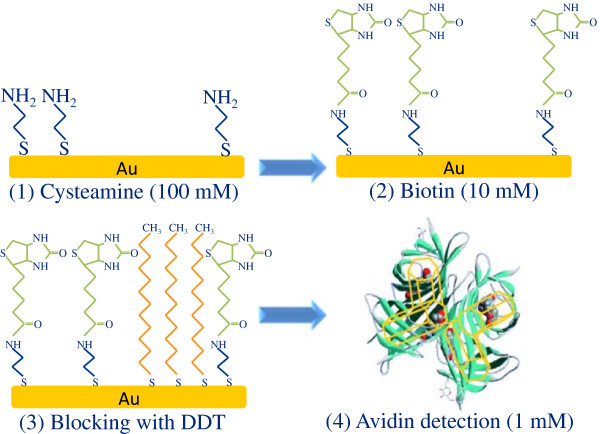
**Sketch of the functionalization process of different molecules.** **(1)** Cysteamine, **(2)** biotin, **(3)** blocking with dodecanethiol, and **(4)** avidin.

Cysteamine, biotin-NHS, dodecanethiol (DDT), avidin, and bovine serum albumin (BSA) were purchased from Sigma Aldrich (St. Louis, MO, USA). The same functionalization procedure was used for GNC (SERS detection) and flat gold film (SPR detection). Figure 2 represents a sketch of functionalization process, where gold surfaces were first functionalized with a cysteamine monolayer, by an overnight incubation in a 100 mM solution of cysteamine in water. The surfaces were then thoroughly rinsed with water. In a second step, the samples were dipped in a 10 mM solution of biotin-NHS in dymethylformamide (DMF) and left to react for 2 h. Afterwards, the sample was thoroughly rinsed first with DMF, then with water. In order to guarantee that no surface was left unfunctionalized, a blocking step was needed. To do that, the nanostructures were soaked in a pure DDT solution for 1 h and then rinsed with ethanol and water. In the SERS experiment, a solution of 1 mM avidin was used to demonstrate the sensing ability of the GNCs.

### Raman spectrum acquisition

Raman spectra were recorded using a Labram spectrophotometer from Horiba Scientific (Kyoto, Japan) for all experiments. The acquisition parameter was fixed to 500 s for avidin/biotin system. A 633-nm laser was used for all experiments with a power of 100 *μ*W. The laser excitation was focused on the substrate using a microscope objective (×80, N.A. = 0.75). The same objective was used to collect the Raman signal from SERS substrates in a backscattering configuration. The Raman spectra were recorded with a spectral resolution of 1 cm ^-1^ and a spatial resolution about 1 *μ*m. For classical Raman measurements in solution, a macro-objective with a focal length of 40 mm (N.A. = 0.18) and a 633-nm laser were used. All obtained spectra have been corrected for acquisition time and laser power so they can be compared.

### SPR measurements

The SPR measurements were recorded using a BIAcore 1990 GE Healthcare system (Pewaukee, WI, USA) with bare gold chips. It was mainly used to test the biotin functionalization procedure and the specificity of this biotin - DDT layer. For this experiment, increasing concentrations of avidin (0, 0.01, 0.03, 0.1, 0.3, 1, 3, 10, 30, and 100 nM) were used to test the surface sensitivity. Furthermore, the functionalized surface was also exposed to a flow of concentrated BSA (1.5 *μ*M) in order to determine the surface specificity for avidin detection.

## Results and discussion

### Gold nanocylinder fabrication

**Figure 3:**
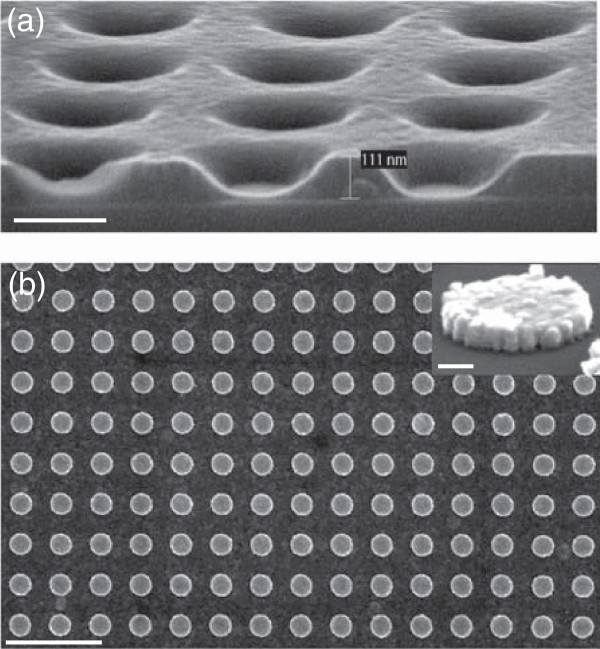
**Gold nanocylinder fabrication by UV-NIL.** **(a)** Imprint in AMONIL resist (scale bar = 200 nm), **(b)** SEM image of the obtained nanocylinders (scale bar = 1 *μ*m), and the inset represents a zoom of a single nanodisk (scale bar = 50 nm).

Firstly, the imprint process is realized in the AMONIL, which is deposited on a PMMA resist. The polymerization is performed with UV exposure (*λ*=365 nm) with 10 mW/cm ^2^ power during time exposure of 20 min. The imprint pressure is 200 mbar. Figure 3a shows a cross section SEM view with nanoholes in the AMONIL resist. The average diameter of holes is around 220 nm, and the residual thickness of AMONIL is around 20 nm. Thus, the AMONIL presence in the hole ground needs a specific RIE process. The removal of the residual AMONIL and the PMMA etch conditions have been reported in reference [17]. During this PMMA etching, we have a good selectivity between the PMMA and AMONIL (*v*_PMMA_/*v*_AMONIL_=2.7). After this etch, a gold thin layer (50 nm) is deposited by electron beam evaporation in order to realize the plasmonic nanocylinders. Previously, an adhesion layer (Cr) for gold is evaporated (3 nm). Figure 3b shows SEM image of gold nanocylinder arrays on 1 mm ^2^. The obtained dimensions are approximately 220 nm for the diameter, 50 nm for the height, and a periodicity of 400 nm. The inset of the Figure 3b is a zoom of a single nanodisk, and we observed a surface roughness relatively important with small tips on the nanodisk border.

### Avidin detection

**Figure 4:**
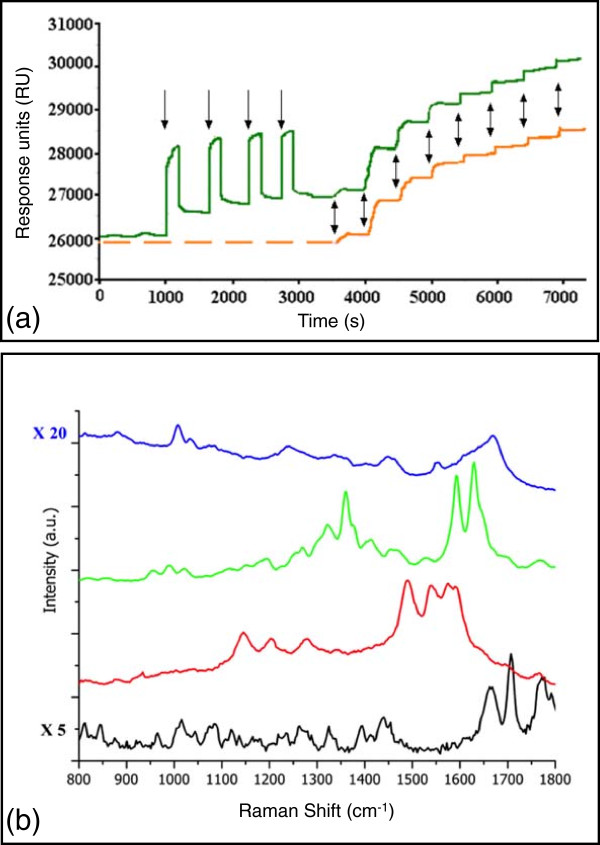
**Optical characterization: SPR and SERS.** **(a)** SPR measurements showing the avidin adsorption onto functionalized gold layer (orange) and after four injections of BSA (green). Single arrows show the BSA injection, and double arrows avidin injections at 0.03, 0.1, 0.3, 1, 3, 10, 30, and 100 nM. **(b)** Raman spectra of biotin-NHS powder multiplied by 5 (black), biotin on GNCs (red), avidin + biotin on GNCs (green), and avidin solution multiplied by 20 (blue).

Avidin detection was performed, thanks to the GNC functionalization with biotin (to capture avidin) and DDT (to block the remaining clean gold surface). In order to validate the functionalization protocol, we have performed SPR measurements on bare gold surface prior to the SERS experiment. Figure 4a presents the result of this experiment. These experiments have been done on the same substrate (bare flat gold surfaces) with two different flow cells. Cysteamine, biotin, and DDT are very light molecules and they cannot be detected by this technique; this is why only the results concerning the adsorption of BSA and avidin are shown. The first curve (orange) shows the result of avidin adsorption on the functionalized gold surface. Increasing concentrations are successively injected followed by a rinse with water, and the result is seen as a curve with a staircase shape. The response units are sensitive to the refractive index of the gold surrounding layer, so that an increase in the response units translates as an increased adsorption of proteins. The first injections (i.e., 0 and 0.01 nM) do not correspond to any step because there are too few avidin molecules immobilized on the surface. For 0.03 nM, a low step is observed; however, this concentration is very low and is not sufficient to fulfill all the biotins present on the surface; 0.1 nM seems to be a good concentration to start the saturation of the biotinylated gold chip. In order to confirm the binding specificity of avidin to biotin, a second experiment was performed. A high concentration of BSA was injected and rinsed four times before the injection of avidin. Although BSA has been injected, the result shows a total recovery of the sensitivity in terms of avidin detection. The functionalization route is thus specific to avidin.

SERS results obtained on biotin and avidin with UV-NIL GNCs are shown in Figure 4b. Since SERS is highly sensitive to the first layer deposited on the gold surface, the red Raman spectrum should then be relevant of the whole functionalization layer (i.e., cysteamine, DDT, and biotin). Yet, cysteamine and DDT have a low Raman cross section; thus, we assume that they would be barely visible and that the red spectrum is mainly due to the biotin molecule in interaction with cysteamine. It is difficult to compare this spectrum with the biotin-NHS powder spectrum. The main reason for this is that the NHS group has been removed during the reaction between biotin and the cysteamine. The main visible peaks in the red spectrum are located at 1145, 1202, 1276, 1493, 1537, 1572, and 1591 cm ^-1^. In a second step, avidin was added onto the sample, giving rise to the green spectrum. A new set of peaks is seen revealing the interaction between avidin and biotin. The spectrum of avidin on biotin is quite different from the one of the avidin solution. Two explanations are given: the interaction between avidin and biotin (this behavior has already been observed in references [22, 23]) and the fact that the biotin-avidin spectrum has been acquired in dry conditions giving rise to probable conformational changes.

In addition, it is more difficult to make an assumption on the number of detected molecules. This is due to the fact that a functionalization layer was used and hypothesis should be made concerning the quality of the functionalization and the association between the molecules. The comparison between the SERS spectra of avidin-biotin and the Raman spectra of an avidin solution (1 mM) and biotin-NHS powder shows that all peaks from the avidin powder are not found on the avidin-biotin SERS spectrum. An explanation is that the SERS experiment has been performed in dry conditions. Conformation changes are probable consequences of drying a protein, and this is reflected in the molecule Raman fingerprint. The second noticeable point is that the avidin and biotin-NHS signals were multiplied, respectively, by 20 and 5 in order to be compared to the SERS spectrum of avidin-biotin (see Figure 4b). In order to check the substrate sensitivity, we have calculated the enhancement factor of the UV-NIL SERS substrate. We compared the SERS signal of avidin-biotin to the Raman signal of 1 mM avidin solution. The number of excited molecules in the SERS experiment is 3.8 ×10^6^ (assuming that the GNC is entirely covered by avidin, that avidin is a sphere with a diameter of 5 nm, and that the collection area is 74.3 *μ*m^2^ for a pattern that contains 1024 nanocylinders). In the Raman experiment, the number of excited molecules is 4.3 ×10^12^. This number is obtained by: *N*_Raman_=*N*_A_*CV*, where *N*_A_=6.023×10^23^ is the Avogadro’s number (mol ^-1^), *C* is the used concentration of Avidin, and *V* is the scattering volume (in L). This scattering volume is defined as follows [24]: *V*=*A*×*H*, where *A*=7.07×10^4^*μ*m^2^ (scattering area = disk area of which the diameter is 300 *μ*m) and *H* is approximately 100 *μ*m (scattering height). If we assume that the peak at 1664 cm ^-1^ is shifted to 1630 cm ^-1^ due to avidin interactions with biotin and GNC, an intensity ratio (*I*_SERS_/ *I*_Raman_) of 32.5 is measured. Then, the enhancement factor (EF) is given by:
1

where *I*_SERS_ and *I*_Raman_ are the SERS and Raman intensities, respectively. *N*_SERS_ and *N*_Raman_ are the number of excited molecules in SERS and Raman experiments, respectively. Thus, we found an EF value of approximately 3.7 ×10^7^ for avidin/biotin system. This EF value is comparable or slightly higher than that obtained by other fabrication techniques such as EBL. Moreover, the advantages of the UV-NIL technique are the fabrication speed, the low cost, reproducibility, and homogeneity of nanostructures on large pattern areas. These advantages are very important parameters for the SERS application. In addition, giving this EF value and considering the signal/noise ratio of the SERS spectrum, it would be possible to dilute the concentration of avidin at least 100 times and still be able to detect it. Thus, we assume that we could detect avidin at concentrations in the micromolar range, which is in agreement with the detection limit already calculated for other proteins but using nanocylinders produced by EBL [11].

## Conclusions

In summary, we have fabricated by the UV-NIL technique a large area of ordered GNCs, which was used as SERS substrate. The use of UV-NIL enabled to reach comparable results with EBL in terms of enhancement factor. Moreover, this UV-NIL technique is faster and less expensive than EBL. The size and shape of nanostructures are homogenous and reproducible on large pattern areas, which are important points for industrial applications of SERS. We detected a weak number of avidin molecules (3.8 ×10^6^) by SERS measurements. A great advantage of SERS is the fingerprint provided by each molecule in order to identify the detected molecules. Finally, the SERS substrates fabricated by UV-NIL have thus showed their ability in biosensing with a good sensitivity.
